# Diffuse large B cell lymphoma CD5-positive arising in an immune deficiency and immune dysregulation setting: A case report and brief review of the literature

**DOI:** 10.1097/MD.0000000000033083

**Published:** 2023-02-22

**Authors:** Miruna Cristian, Radu Andrei Baz, Andreea Georgiana Stoica, Mariana Așchie, Maria Mihaela Ghinea, Mariana Deacu, Madalina Boșoteanu, Anca Florentina Mitroi, Nicolae Dobrin, Ionut Eduard Iordache, Gabriela Izabela Bălțătescu

**Affiliations:** a Faculty of Medicine, “Ovidius” University of Constanta, Constanta, Romania; b Center for Research and Development of the Morphological and Genetic Studies of Malignant Pathology - CEDMOG, “Ovidius” University of Constanta, Constanta, Romania; c Department of Clinical Pathology, “Sf. Apostol Andrei” Emergency County Hospital, Constanta, Romania; d Academy of Medical Sciences, Bucharest, Romania; e Department of Radiology, “Sf. Apostol Andrei” Emergency County Hospital, Constanta, Romania; f Department of Hematology, “Sf. Apostol Andrei” Emergency County Hospital, Constanta, Romania; g Department of Surgery, “Sf. Apostol Andrei” Emergency County Hospital, Constanta, Romania.

**Keywords:** CD5 antigens, diffuse, germinal center, immunohistochemistry, immunological deficiency syndromes, large B cell, lymphoma

## Abstract

**Rationale:**

In the era of antiretroviral therapy, lymphoma is the primary cause of cancer-related death among human immunodeficiency virus (HIV)-infected people and the most prevalent and aggressive non-Hodgkin lymphoma is diffuse large B cell lymphoma, which usually has an aggressive clinical course. CD5-positive diffuse large B cell lymphoma (DLBCL) is an insufficiently studied, relatively new entity, which accounts for 5% to 10% of the DLBCL population. The current study presents the clinicopathological features, diagnostic approach, and clinical outcomes of this HIV-related lymphoma and highlights the importance of the early diagnosis of CD5-positive DLBCL.

**Patient concerns:**

We present a case of a 30-year-old male patient, with a medical history of HIV-positive serology and antiviral treatment, presenting with diffuse abdominal pain and symptoms related to obstruction or perforation, followed by exploratory laparotomy and surgical resection of the small intestine with other areas of involvement. The surgical specimen was morphologically evaluated and immunohistochemical stained.

**Diagnoses and Interventions:**

Histopathologic examination revealed a diffuse neoplastic proliferation of large B lymphocytes within the small intestine, lacking features of other defined types of large B cell lymphoma. The diagnosis of CD5-positive DLBCL subtype was made after immunostaining with twelve monoclonal antibodies (CD3, CD5, CD10, CD20, CD23, CD30, CD68, Cyclin D1, MUM1, Bcl2, Bcl6, and Ki-67). The expression profile of immunohistochemical markers (CD10, Bcl6, and MUM1) established the cell of origin of this case of DLBCL by using the Hans algorithm.

**Lessons:**

The current report highlights the importance of early diagnosis of CD5-positive DLBCL because of its poor prognosis and calls attention to the critical importance to identify immunodeficiencies because doing so affects the types of treatments available. Although cell-of-origin is useful for predicting outcomes, the germinal center B cell like and activated-B cell like subtypes remain heterogeneous, with better, and worse prognostic subsets within each group.

## 1. Introduction

The most prevalent and aggressive non-Hodgkin lymphoma (NHL) is diffuse large B cell lymphoma (DLBCL) NHL. It is most common in older people, usually has an aggressive clinical course, and requires prompt medical treatment.^[1]^

CD5-positive DLBCL remains an insufficiently studied, relatively new entity, which accounts for 5% to 10% of the DLBCL population.^[2]^ In contrast to CD5-negative DLBCL, which is more common in men (1.4: 1), patients with CD5-positive DLBCL have an average age of 70 years and a moderate female predominance (1: 1.2).^[3]^ The international prognostic index (IPI) score is high in the majority of individuals with CD5-positive DLBCL.^[4–6]^

There is debate about whether CD5-positive DLBCL is a distinct clinical entity or merely an immunophenotypic variety of DLBCL with poor prognostic characteristics. Relatively few literature findings suggest worse clinical outcomes in individuals with CD5-positive DLBCL due to resistance to treatment.^[3]^ The lack of understanding in this area encourages more investigation into this particular type of lymphoma.

Lymphoid proliferations and lymphomas associated with immune deficiency and dysregulation are heterogeneous groups of lesions with variable clinicopathologic features.

The World Health Organization classification recognizes 5 types of immunodeficiency-associated lymphoproliferative disorders (LPD) in the 5^th^ Edition of Haematolymphoid Tumours (2022): hyperplasias arising in immune deficiency/dysregulation, polymorphic LPD arising in immune deficiency/dysregulation, Ebstein Barr virus (EBV)-positive mucocutaneous ulcer, lymphomas arising in immune deficiency/dysregulation and inborn error of immunity-associated lymphoid proliferations and lymphomas.

Lymphomas arising in the immune deficiency and immune dysregulation setting (IDD-Lymphomas) may present in any immune deficiency or immune dysregulation setting and cover a spectrum of morphologies including DLBCL. Since diagnostic criteria have been subject to change and are inconsistent across IDD settings in prior classifications, published data on epidemiological aspects of IDD-lymphomas are difficult to compare.^[7–9]^

IDD-lymphomas may involve lymph nodes and extra nodal sites including an allograft.^[7]^ Extra nodal presentation is generally more frequent compared to similar lymphomas in immunocompetent patients.^[10–15]^ The morphologic presentation as DLBCL, or other lymphomas and the time to development of lymphoma largely depends on the severity of immunodeficiency and the particular dysregulations of specific immune cell populations. In the human immunodeficiency virus (HIV) setting, people living with HIV (PLWH) often present at an advanced clinical stage. In the era of antiretroviral therapy (ART), lymphoma is the primary cause of cancer-related death in HIV-infected people Lactate dehydrogenase (LDH) is usually markedly elevated. Moreover, changes in clinical management such as the development of more effective combination ART in PLWH and newer pretransplantation conditioning and induction regimens in posttransplantation settings, have impacted epidemiological features. In the HIV setting the incidence of lymphoma is reportedly increased 60 to 200 times in PLWH.^[7]^

DLBCL arising in the IDD setting are indistinguishable histologically from DLBCL, NOS, or EBV + DLBCL, NOS and the diagnostic criteria are identical except for the critical clinical setting of underlying IDD. The presence of diffuse sheets of large B cells supports the diagnosis of DLBCL. In some patients with IDD, a T/histiocyte-rich microenvironment may be prominent resembling T cell/histiocyte-rich large B cell lymphoma. Lymphomas with angiotropism and angiodestruction with necrosis in an IDD context may be classified as diffuse large B cell lymphoma, lymphomatoid granulomatosis-type, and further reported according to the guidelines for LPD arising in the immune deficiency and immune dysregulation setting.^[7]^

The main purpose of this current study is to report a case of a young male patient, known with HIV infection, following antiretroviral therapy, who presented with gastrointestinal symptoms, and high levels of LDH at diagnosis, who was found to have DLBCL CD5-positive upon resection of the small intestine.

The current report calls attention to the critical importance to identify immunodeficiencies because doing so affects the types of treatments available. A universal framework would make it easier to gain useful biological insights and pave the path for ongoing research in the field, hence a reevaluation of immunodeficiency-associated LPD is urgently needed.

## 2. Case Report

### 2.1. Clinical findings

**Figure 1.**
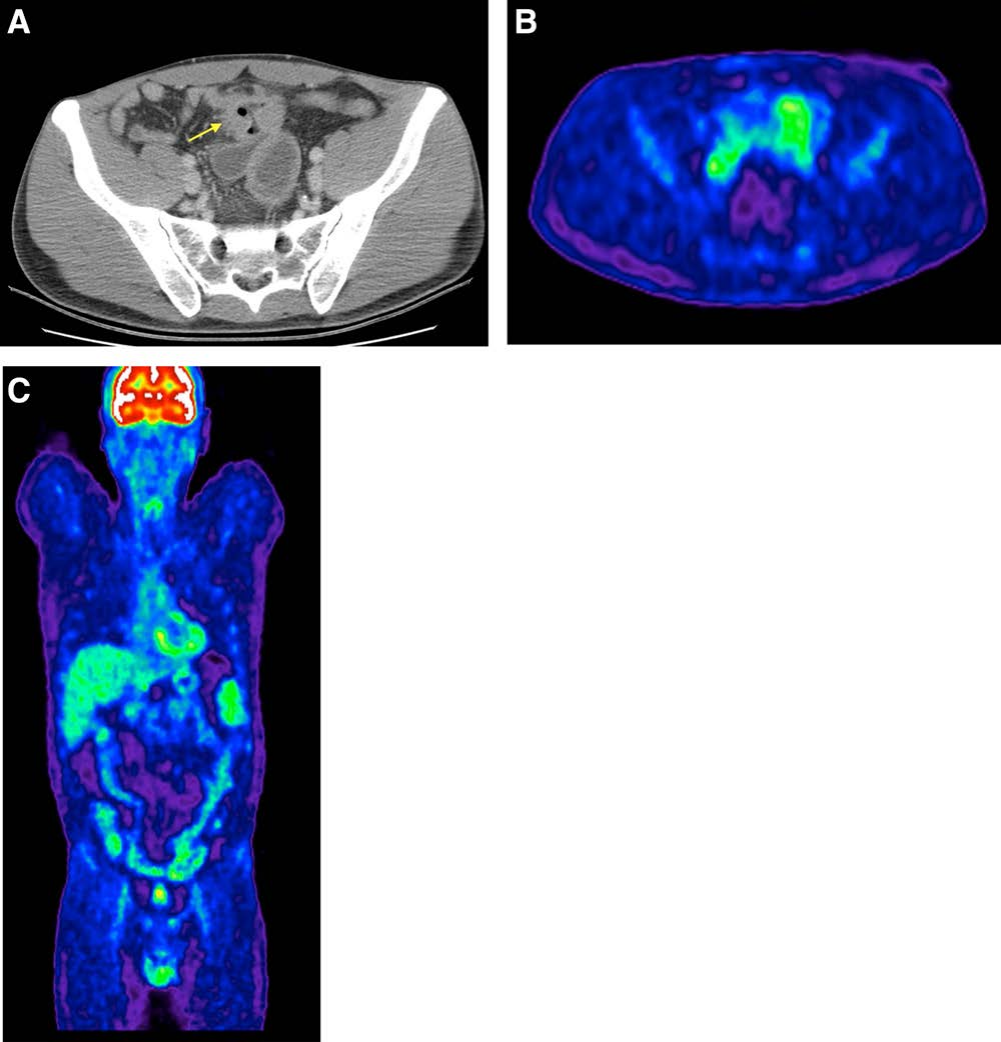
Abdominal CT scan and PET-CT. (A) Abdominal CT scan at the first diagnosis showed circumferential ileum wall thickening involving a short segment of a single bowel loop (arrow). (B), (C) Monitoring PET-CT after the haemato-oncological diagnosis: demonstrates increased metabolic activity at the level of a pelvic ileal loop. CT = computed tomography.

A 30-year-old male patient, known with HIV infection (since the age of 7), under antiretroviral therapy, was admitted to the hospital in June 2019, accusing symptoms of nausea, vomiting, abdominal pain, and lack of bowel transit for gas and fecal matter for about 4 days. The patient was complaining of nonspecific abdominal pain and a lower gastrointestinal endoscopy was performed; results came back negative so a computed tomography (CT) enterography was ordered. Our patient underwent the multi-detector computed tomography scan after drinking about 1.5 L of neutral oral contrast over 45 minutes. Adequate luminal distension was obtained. Scanning protocol included a noncontract phase, an enteric phase (45–the 50 seconds), and a late venous phase (70–80 seconds). The main finding was a significant circumferential ileum wall thickening with homogenous enhancement (Fig. 1A) involving a short segment of a single bowel loop. No fistulas or abscesses were reported. Surgical intervention was performed by practicing segmental resection of the small intestine with a double-layer isoperistaltic later enteral anastomosis and left colectomy, the postoperative diagnosis being mechanical intestinal occlusion by a small bowel tumor with invasion into the bladder and sigmoid. The enlarged spleen was also noted by the surgical team.

The patient was thus admitted to the hematology department. Clinically, the patient was in good condition, Eastern Cooperative Oncology Group performance status 0, stable, normal weight. Physical examination showed an iliac anus, palpable spleen, and no lymphadenopathy. Blood work showed microcytic hypochromic anemia (Hb = 11,2 g/dL), ferritin 5,5 ng/mL, LDH 300U/L, liver and kidney functions tests were normal; hepatitis B and C testing were negative; HIV RNA viral load 84 copies/mL, CD4-T 119 cells/mL, CD8-T 1175 cells/mL, CD4/CD8 = 0,1. The diagnosis of extra nodal HIV-related nongerminal center B cell like (non-GCB) DLBCL, stage IV, IPI 3, central nervous system-IPI 3 was made.

The patient receives counseling about the potential effect of treatment on fertility and options for fertility-preserving measures.

In September 2019 positron emission tomography/CT was performed and showed increased metabolic activity at the level of a pelvic ileal loop (increased FDG uptake), without associated occlusive or subocclusive phenomena (Fig. 1. B and C).

The treatment was started with chemotherapy (CHOP regimen - cyclophosphamide, doxorubicin, vincristine, prednisone) and concomitant administration of ART along with prophylaxis for Pneumocystis jirovecii pneumonia. After 6 cycles of chemotherapy, we performed another positron emission tomography-CT examination with normal ileal metabolic uptake, which suggested a complete response to treatment. The patient was considered disease-free after periodic clinical and paraclinical testing. In July 2020 he is referred to the surgery clinic to remove the iliac anus and restore digestive continuity.

### 2.2. Histopathological examination

**Figure 2.**
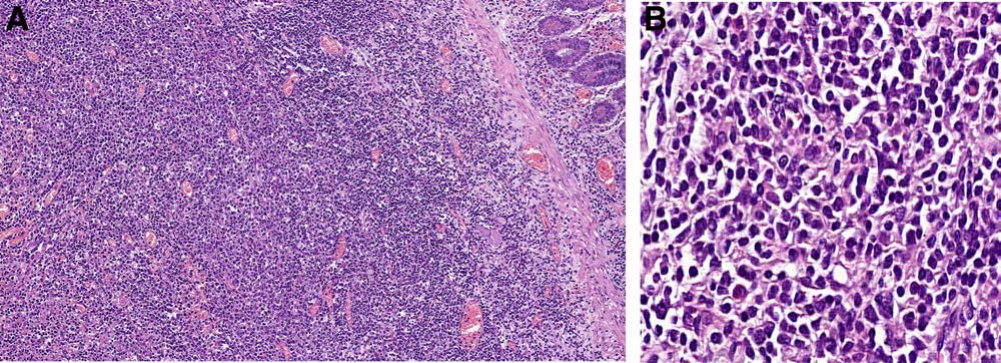
Diffuse large B-cell lymphoma (HE, 40X; HE, 100X), (A, B). The images demonstrate a malignant lymphoid proliferation, with intermediate- to large-sized cells, that are arranged in a diffuse pattern, with a variable amount of cytoplasm, round to oval nuclei, fine to vesicular chromatin, and high mitotic rate.

Macroscopically, 1 ulcerated mass lesion of 3 × 2,1 × 1,4 cm presenting in the enteral mucosa, with adherence to the surrounding large bowel loop. The cut surface reveals a homogeneous, whitish, and fleshy appearance. After the gross examination, the selected surgical specimens were fixed in 10% formalin and paraffin-embedded, then stained with Hematoxylin Eosin (HE). Microscopic examination (Fig. 2. A and B) showed the small intestinal lesion to consist of malignant lymphoid proliferation, with intermediate to large sized cells, that are arranged in a diffuse pattern, with a variable amount of cytoplasm, round to oval nuclei, fine to vesicular chromatin, and high mitotic rate. Areas of necrosis were identified.

One mesenteric lymph node showed involvement by the same lymphoid cells noted in the small intestine. Ileal and colonic resection margins were free. The slides were evaluated by a Nikon Eclipse E600 microscope and representative photos were taken from digital whole slide images, obtained with Huron TISSUEScopeTM 4000XT scanner.

### 2.3. Immunohistochemistry evaluation

**Figure 3.**
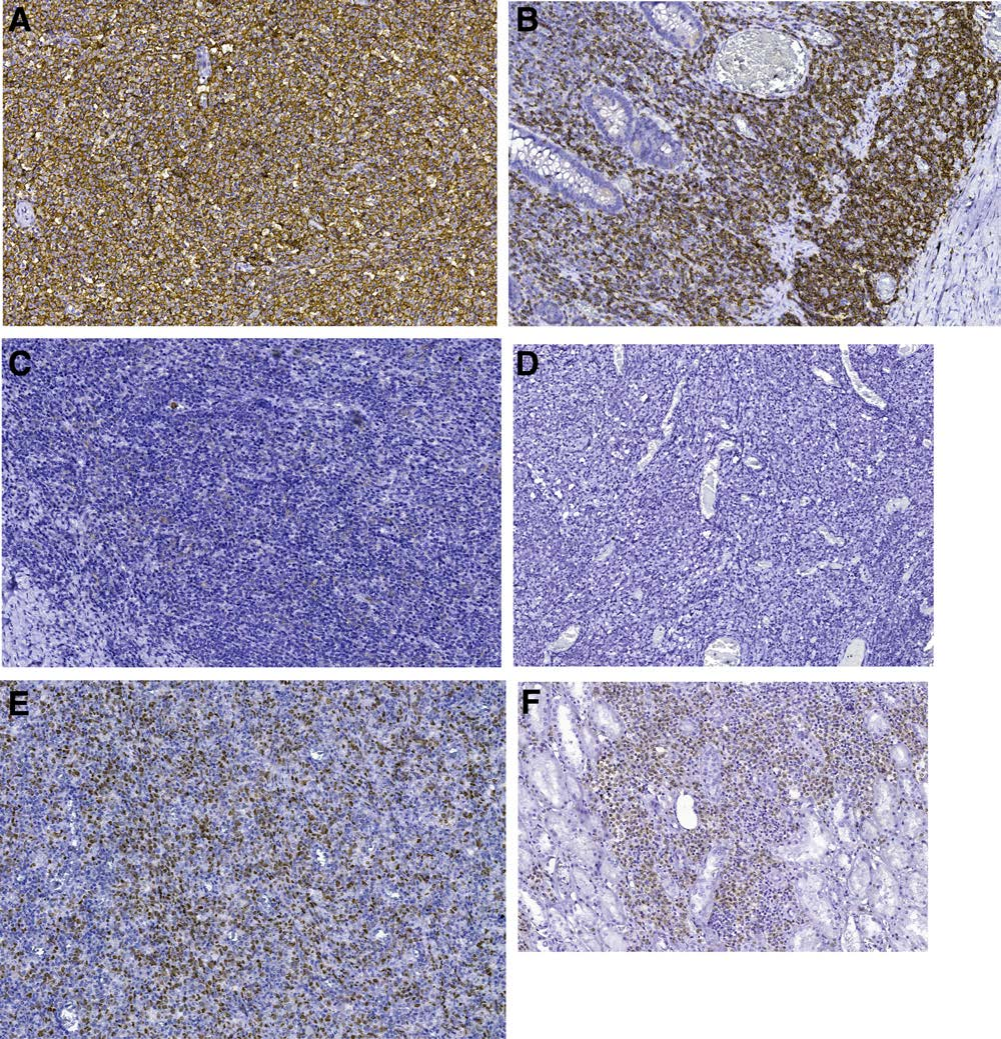
Immunohistochemistry evaluation and determination of the cell of origin of the surgical specimen. (A) The image shows a 95% positive immunostain for the CD20 biomarker (IHC; 40X). (B) CD5 biomarker was positive in scattered cells within the tumor (IHC; 40X). (C) Negative immunostain for CD10 biomarker (IHC; 40X). (D) Negative immunostain for EBV biomarker (IHC; 40X). (E) The image shows focally positive cells for the Bcl-6 biomarker (IHC; 40X). (F) MUM-1 biomarker was positive in scattered cells within the tumor (IHC; 40X).

Further to the histopathological examination, immunohistochemical testing was mandatory, to establish the proliferation line. Immunohistochemical evaluation (Fig. 3) was performed on 4-μm thick sections of a representative formalin fixed, paraffin embedded tissue block from the enteral lesion samples. After epitope retrieval, tissue sections were incubated with a panel of twelve monoclonal mouse antibodies, ready to use from BIOCARE Medical (Table 1).

**Table 1:** Antibodies used for immunohistochemical evaluation.

Antibody	Isotype	Clone	Antigen retrieval	Dilution	External control
Ki67	IgG	SP6	Ki-67	1:50	tonsil or breast cancer
CD20	IgG2a/kappa	L26	CD20 (B-cell)	1:100	tonsil or B-cell lymphoma
CD3	IgG2a	PS1	CD3 (T cell)	1:50	tonsil or T cell lymphoma
CD5	IgG1/kappa	4C7	CD5	1:100	mantle cell lymphoma
CD10	IgG1	56C6	CD10	1:100	tonsil or kidney
CD23	IgG1	1B12	CD23	1:100	follicular lymphoma or tonsil
CD30	IgG2a	CON6D/B5	CD30	1:100	hodgkin or anaplastic large cell lymphoma
CD68	IgG1/kappa	KP1	CD68	1:100	tonsil
Cyclin-D1	IgG	EP12	Cyclin D1	1:100	mantle cell lymphoma and breast cancer
BCL-2	IgG1/kappa	100/D5	Bcl-2a	1:100	follicular lymphoma or tonsil
BCL-6	IgG2b	LN22	AA 1-350 N-terminus human Bcl-6 oncoprotein	1:100	tonsil or follicular lymphoma
MUM-1	IgG	BC 5	MUM-1 protein	1:300	tonsil

In the present study, immunophenotyping showed the intermediate- to large-sized cells to be of B cell origin with strong CD20 (Fig. 3A) and Bcl-6 (Fig. 3E) positive; CD5 was positive in scattered cells within the tumor (Fig. 3B) and focally positive cells for MUM-1 biomarker (Fig. 3F) were observed. Negative results were noticed for CD3, CD10 (Fig. 3C), CD23, CD30, Cyclin D1 and EBV (Fig. 3D) biomarkers. The Ki67 proliferation index is high.

Based on the morphology and immune profile, a diagnosis of diffuse large B-cell lymphoma, CD5-positive, with centroblasts morphology.

Using the Hans algorithm, we concluded that the cell of origin was a non-GCB.

## 3. Discussions

According to Levine A.M., the incidence of 3 malignancies has increased in conjunction with the epidemic of HIV disease, and they have currently considered acquired immunodeficiency syndrome (AIDS)-defining conditions. One of these AIDS-defining conditions is intermediate or high-grade B cell lymphoma, which became AIDS-defining in 1985. The above-mentioned study proved that approximately 40% of all patients with AIDS have developed cancer during the course of HIV infection.^[16]^ Further, as survival has improved in HIV disease, the incidence of these malignancies has increased.^[16]^

In the study of International Collaboration on HIV and Cancer, Highly Active Antiretroviral Therapy and Incidence of Cancer in Human Immunodeficiency Virus-Infected Adults they demonstrated that the sharp decline in the incidence of Kaposi sarcoma and NHL and the lack of an increase in other malignancies since the widespread use of highly active antiretroviral therapy is reassuring for HIV-infected subjects and do not support the view that cancer incidence rates might increase as HIV-infected people survive longer.^[17]^ In the present report, we add new insights to this view by presenting an HIV-infected patient that had survived longer with highly active antiretroviral therapy, but after several years he developed an AIDS-related lymphoma.

According to Bellan et al^[18]^, since the beginning of the HIV pandemic, a strong correlation between HIV infection and the occurrence of a particular subset of malignancies has been recognized and the incidences of some AIDS-defining cancers have decreased significantly as a result of the development of highly active antiretroviral therapy. This shows that HIV may play both a direct and indirect function in developing tumors linked to the virus.^[18]^

Globally, earlier studies showed that, since the beginning of the HIV pandemic, more patients with AIDS-related lymphoma have been diagnosed.

According to a retrospective study by Gopal et al^[19]^, in the era of ART, lymphoma is the primary cause of cancer-related death in HIV-infected people. The above-mentioned study proved that, of 23 050 HIV-infected individuals, 476 (2.1%) developed lymphoma, and 201 [42.2%] individuals developed DLBCL from 1996 to 2010.^[19]^

According to Jain et al^[20]^, CD5 positivity is classically associated with a poor prognostic factor, having female preponderance, more bone marrow involvement, higher LDH levels, B symptoms on presentation, and Ann Arbor stage 3 to 4 on diagnosis.^[21]^ In this current case, based on the morphology and immunophenotype, we present a case of a young male patient with a diagnosis of AIDS-related diffuse large B cell lymphoma, CD5-positive, with a high LDH level and stage IV Ann Arbor at presentation. Using the Hans algorithm, we concluded that the cell of origin was a non-GCB. Various DLBCL subtypes have been shown to have different prognostic implications, including germinal center B cell like and non-GCB.^[22]^ The resistance of non-GCB DLBCL to chemotherapy and the resultant poor prognosis is thought to be due to the constitutive activation of NF-κB and its antiapoptotic target genes.^[23,24]^

In addition, each presentation has its defining characteristic, of which CD5 positivity is notoriously known for being a poor prognostic factor.^[21]^ Our patient was considered to have a good outcome, being considered disease free after periodic clinical and paraclinical testing, and being referred to the surgery clinic for removal of the iliac anus and restoration of digestive continuity.

## 4. Conclusion

In this study, we described a case of a young male patient, diagnosed based on the morphology and immunophenotype, with an AIDS-related diffuse large B cell lymphoma, CD5-positive, non-GCB, in stage IV of disease (Ann Arbor) with IPI 3 and central nervous system-IPI 3, with a good outcome after chemotherapy.

In conclusion, less immunosuppression and better HIV control at diagnosis characterize the varied, evolving nature of HIV-associated lymphoma, and a deeper understanding of biology is required to enhance outcomes in light of stable survival and increased death for lymphoma patients using ART.

## Acknowledgements

This research was performed in the Center for Research and Development of the Morphological and Genetic Studies of Malignant Pathology from the “Ovidius” University of Constanţa.

## Author contributions

**Conceptualization:** Miruna Cristian, Andreea Georgiana Stoica, Mariana Deacu, Gabriela Izabela Bălțătescu.

**Data curation:** Miruna Cristian, Radu Andrei Baz, Andreea Georgiana Stoica, Anca Florentina Mitroi, Gabriela Izabela Bălțătescu.

**Formal analysis:** Andreea Georgiana Stoica, Madalina Boșoteanu, Anca Florentina Mitroi, Nicolae Dobrin.

**Investigation:** Miruna Cristian, Andreea Georgiana Stoica, Mariana Deacu, Maria Mihaela Ghinea, Ionut Eduard Iordache.

**Methodology:** Miruna Cristian, Andreea Georgiana Stoica, Mariana Deacu, Maria Mihaela Ghinea, Madalina Boșoteanu, Nicolae Dobrin, Gabriela Izabela Bălțătescu.

**Project administration:** Miruna Cristian, Mariana Așchie, Nicolae Dobrin.

**Resources:** Miruna Cristian.

**Supervision:** Mariana Deacu, Maria Mihaela Ghinea, Madalina Boșoteanu, Anca Florentina Mitroi, Gabriela Izabela Bălțătescu.

**Validation:** Radu Andrei Baz, Mariana Așchie, Mariana Deacu, Maria Mihaela Ghinea, Madalina Boșoteanu, Gabriela Izabela Bălțătescu.

**Visualization:** Miruna Cristian, Andreea Georgiana Stoica, Mariana Așchie.

**Writing – original draft:** Miruna Cristian, Andreea Georgiana Stoica.

**Writing – review & editing:** Miruna Cristian, Radu Andrei Baz, Andreea Georgiana Stoica, Mariana Așchie, Mariana Deacu, Maria Mihaela Ghinea, Madalina Boșoteanu, Anca Florentina Mitroi, Gabriela Izabela Bălțătescu.
